# Remote postconditioning ameliorates stroke damage by preventing let-7a and miR-143 up-regulation

**DOI:** 10.7150/thno.48135

**Published:** 2020-10-27

**Authors:** Antonio Vinciguerra, Pasquale Cepparulo, Serenella Anzilotti, Ornella Cuomo, Valeria Valsecchi, Salvatore Amoroso, Lucio Annunziato, Giuseppe Pignataro

**Affiliations:** 1 Division of Pharmacology, Department of Neuroscience, School of Medicine, “Federico II” University of Naples, Via Pansini, 5, 80131, Naples, Italy;; 2 IRCCS SDN Napoli, Italy;; 3 Department of Biomedical Sciences and Public Health, School of Medicine, Università “Politecnica delle Marche”, Via Tronto 10/A, Ancona, 60126, Italy

**Keywords:** stroke, remote limb postconditioning, microRNA, let-7a, miR-143

## Abstract

Remote limb ischemic postconditioning (RLIP) is a well-established neuroprotective strategy able to protect the brain from a previous harmful ischemic insult through a sub-lethal occlusion of the femoral artery. Neural and humoral mechanisms have been proposed as mediators required to transmit the peripheral signal from limb to brain. Moreover, different studies suggest that protection observed at brain level is associated to a general genetic reprogramming involving also microRNAs (miRNAs) intervention.

**Methods:** Brain ischemia was induced in male rats by transient occlusion of the middle cerebral artery (tMCAO), whereas RLIP was achieved by one cycle of temporary occlusion of the ipsilateral femoral artery after tMCAO. The expression profile of 810 miRNAs was evaluated in ischemic brain samples from rats subjected either to tMCAO or to RLIP. Among all analyzed miRNAs, there were four whose expression were upregulated after stroke and returned to basal level after RLIP, thus suggesting a possible involvement in RLIP-induced neuroprotection. These selected miRNAs were intracerebroventricularly infused in rats subjected to remote ischemic postconditioning, and their effect was evaluated in terms of brain damage, neurological deficit scores and expression of putative targets.

**Results:** Twenty-one miRNAs, whose expression was significantly affected by tMCAO and by tMCAO plus RLIP, were selected based on microarray microfluidic profiling. Our data showed that: (1) stroke induced an up-regulation of let-7a and miR-143 (2) these two miRNAs were involved in the protective effects induced by RLIP and (3) HIF1-α contributes to their protective effect. Indeed, their expression was reduced after RLIP and the exogenous intracerebroventricularly infusion of let-7a and miR-143 mimics prevented neuroprotection and HIF1-α overexpression induced by RLIP.

**Conclusions:** Prevention of cerebral let-7a and miR-143 overexpression induced by brain ischemia emerges as new potential strategy in stroke intervention.

## Introduction

Due to the complexity of the pathology, stroke continues to be an unmet medical need. In the last decades, targeting single genes or single pathways for therapeutic intervention has failed in its translation from bench to bedside 1. Therefore, a prominent role is being attributed to multitarget strategies, eventually endogenously activated 2-4. Among them a category of small non-coding RNA, namely microRNAs (miRNAs), have largely shown the capacity to regulate large set of evolutionary conserved coding genes 5, thus representing a novel approach of gene therapy with an increasing involvement in multifactorial neurological disorders. miRNAs are a large class of non-coding RNA, composed of 20-22 nucleotides, involved in the regulation of post-transcriptional stability of RNA messenger transcripts 6,7. Several miRNAs are expressed in a tissue-specific manner and their expression patterns are reflective of underlying physiologic and pathologic processes and may mediate protective effects endogenously activated. In fact, the failure of numerous stroke clinical trials has prompted researchers to identify alternative mechanisms of protection. It is well known that different kinds of living organisms, exposed to environment alteration, can acquire transient tolerance to these changes, otherwise lethal 8. A well-documented explanation lies in the activation of cellular response mechanisms induced by sub-toxic stimuli and involving a genomic reprogramming 9. This phenomenon has been described for all organs of a wide variety of species, from bacteria to mammalian cells 10.

In the last years, several groups of investigators have directed considerable attention toward evidence that a short occlusion of a distant artery, such as femoral artery, is able to protect an organ undergoing an ischemic insult, a phenomenon called remote ischemic conditioning (RIC) 11-13. It is reasonable to consider that, when a sub-toxic ischemic episode is applied in a distant organ after a longer harmful ischemia, endogenous humoral and neural pathways are recruited both at the blood level and in the damaged brain tissue so as to give neuroprotection. Therefore, it is conceivable to hypothesize the existence of a cross-talk between periphery and brain where a crucial role seems to be mediated by the release of humoral factors including small non-coding RNAs, which may locally activate nerve afferents or enter the systemic circulation 14. In fact, recent data have revealed that miRNAs represent valuable tools as biomarkers and as potential disease-modifying agents 15. Moreover, based on their ability to regulate gene expression, microRNAs can contribute to enhance the activation of mechanisms of brain recovery and repair in ischemic stroke patients 16.

Considering these premises, the purpose of the present study was to characterize the peculiar signature of miRNAs modulated within the brain by remote limb ischemic postconditioning (RLIP) and involved in RLIP-induced neuroprotection in order to propose new miRNA modulator agents as possible strategy in stroke intervention.

## Methods

### Animals

100 male Sprague-Dawley rats (Charles River), weighting 200 g to 250 g, were housed under diurnal lighting conditions (12 h darkness/light) and in a conditioned room (23°C). Experiments were performed according to the international guidelines for animal research and approved by the Animal Care Committee of “Federico II”, University of Naples, Italy. Animals, during any surgical or invasive procedure, were anesthetized using a mixture of oxygen and sevoflurane at 3.5% (Medical Oxygen Concentrator LFY-I-5A), and the rectal temperature was maintained at 37±0.5 °C with a heat-controlled mat (Harvard Apparatus). Among 100 animals used for the present study, 19 have been excluded from the statistical analysis. In particular, 6 died during the surgical procedures and 13 were excluded because the surgical procedure was not successful/ the drug delivery system failed.

### Transient focal ischemia

Stroke was performed in rats by transient middle cerebral artery occlusion (tMCAO), a surgical procedure, modified and readapted in our laboratory 17, that consists of the insertion of a suture filament into the internal carotid artery until the MCA origin 18. Briefly, under an operating stereomicroscope (Nikon SMZ800, Nikon Instruments, Florence, Italy), the right carotid bifurcation was carefully exposed, and the external carotid artery (ECA) was coagulated distal to the bifurcation. A silicon-coated nylon filament (Doccol Co) was inserted through the ECA stump and gently advanced (19 mm) into the right internal carotid artery until it blocked the origin of the MCA. The surgical wound was closed and the filament was left in place. After 100-min of MCA occlusion, the filament was gently withdrawn to restore blood flow. In order to expose the animals to the same concentration of gaseous anesthesia, all animals were kept under anesthesia for 3 h. Achievement of ischemia was confirmed by monitoring regional cerebral blood flow in the area of the right MCA. Cerebral blood flow (CBF) was monitored through a disposable microtip fiber optic probe (diameter 0.5mm) connected through a master probe to a laser Doppler computerized main unit (PF5001; Perimed) and analyzed using PSW Perisoft 2.5 19. Once a stable CBF signal was obtained, the MCA was occluded. CBF monitoring was continued up to 30 min after the end of the surgical procedure, when the occurred reperfusion was verified. Animals that did not show a CBF reduction of at least 70% were excluded from the experimental group, as were animals that died after ischemia induction.

Animals were constantly monitored even in the intermediate time between starting surgical procedures and a 24-h primary endpoint according to our protocol. Before proceeding with the evaluation of neurological deficits, all animals were weighed. Excessive body weight loss or any sign of excessive suffering were considered criteria for excluding animals from the experimental design. In addition, only animals showing a 70% reduction in cerebral blood flow (Laser Doppler analysis) were included in the experimental groups.

To measure arterial blood gases before and after ischemia induction, a catheter was inserted into the femoral artery and a small amount of blood withdrawn and analyzed (Rapid lab 860; Chiron Diagnostic, Medfield, MA, USA). No differences between the examined experimental groups were detected in PaO_2_, PaCO_2_, and pH mean values (Data not shown).

### Remote limb ischemic postconditioning

Remote limb ischemic postconditioning (RLIP) was induced by subjecting ischemic animals to a brief cycle of femoral artery occlusion (FAO), as previously described 20. Briefly, after 20 min of reperfusion from tMCAO, femoral artery was identified, isolated and occluded with two microserrafine clips (Fine Science Tools) to stop the blood flow for a duration of 20 min. Immunohistochemical analysis revealed that in all experimental groups, FAO induced no damage to the region supplied by the femoral artery 20.

### Evaluation of the Infarct Volume and Neurological Deficits

Animals were all killed by decapitation 24 h after ischemia. Brains were quickly removed, sectioned coronally at 1 mm intervals, and stained by immersion in the vital dye (2%) 2,3,5-triphenyltetrazolium hydrochloride (TTC). The infarct volume was calculated by summing the infarction areas of all sections and by multiplying the total by slice thickness. To avoid that edema could affect the infarct volume value, it has been chosen to express the infarct volume as percentage of the infarct, calculated by dividing the infarct volume by the total ipsilateral hemispheric volume 21-23. Neurological scores were evaluated 24 h after reperfusion according to 2 scales: a general neurologic scale and a focal neurologic scale. In the general score,the following 6 general deficits were measured: (1) hair conditions, (2) position of ears, (3) eye conditions, (4) posture, (5) spontaneous activity, and (6) epileptic behavior. For each of the 6 general deficits measured, animals received a score ranging between 0 and 12 depending on the severity of signs. The scores of investigated items were then summed to provide a total general score. In the focal score, the following 7 areas were assessed: (1) body symmetry, (2) gait, (3) climbing, (4) circling behavior, (5) front limb symmetry, (6) compulsory circling, and (7) whisker response. For each of these items, animals were rated between 0 and 4 depending on severity. The 7 items were then summed to give a total focal score. Infarct volumes and neurological scores were evaluated in a blinded manner by individuals who did not perform the surgical procedures.

### Drugs and Chemicals

To explore the effect of in vivo overexpression of selected miRNAs, miRNA mimics (miRCURY LNA miRNA Mimic, Qiagen) were used. All mimics, purchased in dried down pellets, were diluted to the final concentration in saline solution (0.9% NaCl) previously filtered (Microglass filters). miRNA mimics administered were the following: hsa-let-7a-5p (YM00470408); rno-miR-143-3p (YM00470034); rno-miR-485-3p (YM00470535); rno-miR-451-5p (YM00471387); Negative Control Mimic (YM00479902), LNA-let-7a (EX00567890), LNA-miR-143 (EX006789011).

### Intracerebroventricular Administration of miRNA Mimics

In rats positioned on a stereotaxic frame, a 23-g stainless steel guide cannula (Alzet Brain Infusion Kit 1) was implanted into the right lateral ventricle using the stereotaxic coordinates of 0.4mm caudal to bregma, 2 mm lateral and 2 mm below the dura^20^. The cannula was fixed to the skull using dental acrylic glue and small screws. All miRNA mimics were intracerebroventricularly (icv) continuously infused (1 µl/hr for 48 h) through an osmotic minipump (model # 1003D Alzet) implanted in a subcutaneous pouch and connected to the lateral ventricles through a plastic tube and a cannula stereotaxically implanted.

The Anti-miRNA (LNA) were icv infused at the concentrations of 10 μM (9 μg/kg body weight) starting 24 h before tMCAO induction 24. Mirna Mimics were icv infused at a concentration of 10 µM (0.6 mg/kg) 25.

### Sample collection and RNA isolation

Brain regions corresponding to the ischemic core and penumbra were dissected 24 h after surgical procedures from rats subjected respectively to sham surgery, tMCAO and tMCAO + RLIP and 3-5 biological replicate samples per group were collected 22. In particular, ischemic core included temporoparietal cortex and the whole ipsilateral striatum, whereas penumbra brain regions included all surrounding brain cortex 26.

Total RNA from brain tissues was extracted with Trizol following supplier's instruction (TRI Reagent® - Sigma) and RNA quality was assessed using a Thermo Scientific™ NanoDrop™ One Microvolume UV-Vis Spectrophotometer. RNA samples were sent to LC Sciences (Houston, Texas, USA), a global biotechnology company that is provided with a microRNA microarray service.

### MicroRNA Expression Profiling by Microarray

miRNA microarray analysis including separation, quality control, labelling, hybridization and scanning was performed by LC Sciences (Houston, TX, USA) on brain samples collected as above. The array contained 810 rat mature miRNA probes for whole rattus norvegicus miRNome based on a database of published miRNA sequences and annotation (Sanger miRbase Release 21.0). In details, hybridization was performed on a µParaflo microfluidic chip using a micro-circulation pump (Atactic Technologies, Inc., Houston, TX, USA). After the hybridization, the chips were washed and then the fluorescence data images were collected using a laser scanner (GenePix 4000B; Molecular Devices, LLC, Sunnyvale, CA, USA) and digitized using Array-Pro image analysis software (Media Cybernetics, Inc., Rockville, MD, USA). The chips were scanned at a pixel size of 10 µM with Cy3 Gain at 460 nm and the Cy5 Gain at 470 nm scanning. The data were analyzed by first subtracting the background and then normalizing the signals using a LOWESS filter (Locally-weighted Regression). Student's t-test analysis was conducted for individual comparisons between two experimental groups, while the one-way analysis of variance (ANOVA) was performed to compare with each other the experimental groups of RLIP, tMCAO and sham. The false discovery rate (FDR) was p < 0.05 and served as the cut-off criteria. The data were log_2_ transformed and median centred by Cluster 3.0 software (Informer Technologies Inc., Los Angeles, CA, USA) and then further analyzed with hierarchical clustering with average linkage (data not shown). An heat maps was generated reporting differences of signal intensities across all samples within each specific miRNA analysed and values were presented in colour coded blocks with green being negative value, black being zero, and red being positive value.

### Validation of Microarray Results by Real-Time Polymerase Chain Reaction

Results obtained by microarray were validated by real-time PCR. For validation experiments, LC Sciences recommended to focus on those miRNAs that show a minimum intensity in Cy5 fluorescence of 1000-2000 in at least one of the sample groups of the in-depth analysis. In custom plates RT-PCR experiments, only miRNAs showing at least a 1.5 fold change (FC) in expression which corresponds to an average log_2_ value > +0.75 or < -0.75 of signals intensities respectively of tMCAO and tMCAO + RLIP compared to sham-operated animals were selected. In addition, to the aforementioned parameter, the selection took into account those miRNAs showing an inverse pattern of expression between tMCAO and tMCAO + RLIP. RNA was extracted by brain tissues with Trizol following supplier's instruction (TRI Reagent® - Sigma). For retrotranscription, High Capacity cDNA Reverse Transcription Kit (Applied Biosystems) was used, following the protocol for Creating Custom RT and Preamplification Pools using TaqMan® MicroRNA Assays. Briefly, 50 ng of RNA were retrotranscribed in cDNA using RT Primer Pool containing a mix for all 21 selected miRNAs and for 4.5S RNA or U6 snRNA, as endogenous control, and incubating the reaction mix at 16 °C for 30 min, 42 °C for 30 min and at 85 °C for 5 min. Then, cDNA product was preamplificated using TaqMan PreAmp Master Mix and appropriate PreAmp Primer Pool mix, and letting react with the following protocol: 95 °C for 10 min, 55 °C for 2 min, 72 °C for 2 min, twelve cycles of two amplification steps, composed of 15 s at 95 °C and 4 min at 60 °C, and finally a phase of 10 min at 99.9 °C. At the end of preamplification 25 µl of DNA were obtained and diluted in 175 µl of 0,1 X TE buffer. Real-time PCR was performed in Taqman Custom Plates (Applied Biosystems) preloaded with TaqMan® MicroRNA Assays (Applied Biosystems, 4427975) of interest on the bottoms of wells using TaqMan® Universal Master Mix II (Applied Biosystems), as described above. Each Custom Plate (of 96 wells) contained all primers for 2 samples (in triplicate): one sample of sham group and one sample of tMCAO or RLIP group. TaqMan probes used were the following: rno-let-7a-5p (ID: 000377); hsa-let-7b-5p (ID: 002619); hsa-let-7c-5p (ID: 000379); hsa-let-7d-5p (ID: 002283); hsa-miR-15b-5p (ID: 000390); rno-miR-21-5p (ID: 000397); hsa-miR-27b-3p (ID: 000409); rno-miR-32-3p (ID: 463582_mat); rno-miR-34c-3p (ID: 002584); rno-miR-103-3p (ID: 000439); hsa-miR-125a-5p (ID: 002198); hsa-miR-126a-3p (ID: 002228); rno-miR-143-3p (ID: 000466); rno-miR-150-5p (ID: 000473); hsa-miR-181c-5p (ID: 000482); hsa-miR-191a-5p (ID: 002299); rno-miR-210-5p (ID: 464011_mat); rno-miR-382-5p (ID: 000572); rno-miR-451-5p (ID: 001141); rno-miR-466b-5p (ID: 002066); rno-miR-485-3p (ID: 462841_mat); miRNA Control Assay 4.5S RNA (ID: 001716); miRNA Control Assay U6 snRNA (ID: 001973).

### Fluorescent In Situ Hybridization (FISH) miRNA Assay

For in situ hybridization all the procedures were performed in autoclaved and RNAse free solutions. AGCTACAGTGCTTCATCTCA was used to detect miR-143-3p and ACTATACAACCTACTACCTCA to detect let-7a-5p (Exiqon). Rats were perfused with 1× phosphate-buffered saline (PBS) and 4% paraformaldehyde solution PBS. The brain tissues were fixed in 4% paraformaldehyde solution PBS overnight at 4 °C and subsequently cryoprotected in 30% sucrose PBS overnight at 4 °C and cryosectioned at 10 μm thickness 27,28. Frozen tissue sections were prepared following the description of MicroRNA Protocol for In situ Hybridization on Frozen Sections (Exiqon, Denmark). Briefly, brain sections were submerged in Neutral buffered Formalin 10% for 15 min e then washed in PBS for 3x 5 min. The sections were incubated in proteinase K buffer containing 1M tris-HCl pH 7.4, 0.5 M EDTA, 5M NaCl, proteinase K 15 μg/mL in RNase-free water, for 10 min at 37 °C then the sections were washed 3 times x 3 min in PBS. Brain slices were then incubated in 3% H_2_O_2_ for 5 min to inhibit endogenous peroxidase activity, and then washed in PBS for 3x 3min. Sections were sequentially hybridized for 1 h at 55° and 54 °C for mir-143-3p (5'DIG and 3'DIG) and mir-let7a-5p (5'DIG and 3'DIG) respectively. The final concentration of probes was 20 nM. The sections were hybridized in hybridization buffer containing: 50% deionized formamide 0.3M NaCl, 20 mM Tris HCL, pH 8.0, 5 mM EDTA, 10mM NaPO4, pH 8.0, 10% Dextran Sulfate, 1x Denhardt's solution, 0.5 mg/mL yeast RNA and probes. Post-hybridization washes were performed sequentially 2x 5 min at hybridization temperature in 5x SSC buffer, 3x 5 min at hybridization temperature in 1x SSC buffer, 2x 5 min at hybridization temperature in 0,2x SSC and 1x 5 min at room temperature in 0,2x SSC buffer. Following the stringent washing, sections were incubated in blocking solution containing: 2% sheep serum, 1% BSA in PBS-0,1% Tween for 15 min at room temperature. Then, the sections were incubated for 60 min with Anti-Digoxigenin-POD, Fab fragments (Roche Diagnostics GmbH, Germany) diluted 1:400 in 1% sheep serum 1% BSA and PBS 0.05% tween. Then, the sections were washed in PBS for 3x 5min and incubated for 5 min in Cy2 conjugated Tyramide (TSATM Plus Fluorescein kit, PerkinElmer, USA) by diluting TSA stock solution 1:50 in 1x Amplification Diluent. After washing 3x 10 min with TBS, sections were incubated for 30 min in 3% H_2_O_2_ in TBS to quench peroxidase activity from the initial TSA reaction. After washing, sections were incubated with the following primary antibodies in blocking solution: anti-NeuN 1:200 (Elabscience, USA) anti-GFAP 1:200 (Millipore, USA) overnight. Sections were sequentially washed 3x 10 min with PBS and then incubated for 2 h in Alexa Fluor 594-conjugated donkey anti-mouse/rabbit antiserum diluted 1:300 in blocking solution 28. Following washing 3x 10 min with PBS, sections were incubated with HOECHST for 20 min and mounted onto slides using Fluoromount™ Aqueous Mounting Medium, (SIGMA, Germany) air-dried and stored in the dark room. As controls, the sections were incubated without the Anti-Digoxigenin-POD or without the TSATM Plus Fluorescein or primary antibodies and the immunoreactivity was completely abolished (data not shown).

### Western Blot

Rat brain samples were homogenized in a lysis buffer (50mM Tris-HCl pH 7.4, 1mM EGTA; 150mM NaCl; 0.5% NP-40; 1mM NaF; 1mM sodium orthovanadate; 0.2% sodium deoxycholate; 1mM PMSF) containing protease and the phosphatase inhibitor. After centrifugation at 20,000g at 4 °C for 15 min, the supernatants were collected. Protein concentration was measured using Bradford method, by means of a spectrophotometer (Eppendorf). Then, 50μg of protein was mixed with a Laemmli sample buffer (5% 2-mercaptoethanol) and heated at 95 °C for 5 min. The samples were resolved by sodium dodecyl sulfate polyacrylamide gel electrophoresis and transferred to nitrocellulose membranes (0.22 µm). Blots were probed with antibodies to HIF1α (1:1000, rabbit monoclonal, Abcam, ab179483), MKP1 (1:500, rabbit polyclonal, Abcam, ab61201), kRAS (1:500, rabbit polyclonal, Abcam, ab196630) and α-tubulin (1:8000, Sigma, T5168) diluted in tris buffered saline (TBS-T) 5% bovine serum albumin (BSA) overnight (4 °C). Then, signals were detected using horseradish peroxidase-conjugated secondary antibody (1:2000; mouse and rabbit Amersham; 60 min at room temperature in TBS-T 5% BSA) and an enhanced luminescence kit (ImmunoCruz, Santa Cruz Biotechnology).

### Statistical Analysis

Values were expressed as means ± standard error of the mean (S.E.M.). Real-Time PCR results were expressed as fold change (2^-ΔΔCt^) compared to the control group settled to 1, following the instructions derived from the literature 29. Briefly, difference between Ct values of gene of interest and internal control (ΔCt) was calculated for both control sample and target sample. Then, difference between ΔCt of target sample and control sample (ΔΔCt) was calculated. Fold change of gene expression of target samples compared to control sample was reported as 2^-ΔΔCt^. Statistical analysis was performed with GraphPad Prism 5.0 (GraphPad Software, Inc., San Diego, CA), using one-way analysis of variance followed by Newman-Keuls post-test for group more than 2. To compare two groups unpaired t-test was used. Statistical significance was accepted at the 95% confidence level (p < 0.05).

## Results

### Remote limb postconditioning induced a different miRNAs expression in brain compared to cerebral ischemia alone

**Figure 1:**
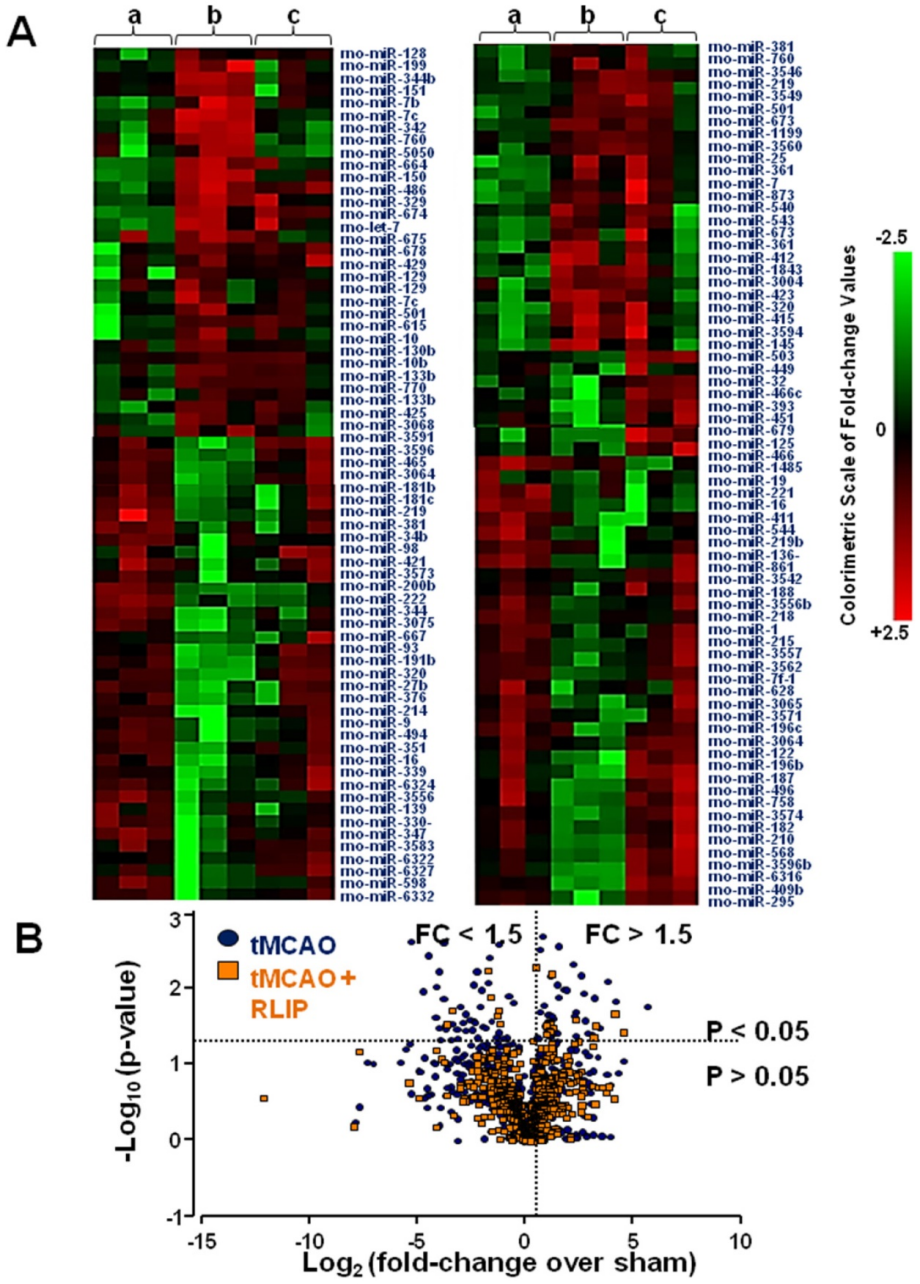
** Heat map of microRNA expression profiles in the whole ischemic area from sham-operated, tMCAO and tMCAO + RLIP animals. (A)** The average of signal intensity values for each significantly expressed miRNA is reported (p < 0.05). Red and green indicate up- and down-regulation respectively, according to the colorimetric scale in the right side of the panel. a, b and c indicate the three experimental group: (a) Sham, (b) tMCAO and (c) tMCAO + RLIP animals. Each vertical line refers to three samples for each experimental group. **(B)** Volcano plot analysis in the lower side of panel shows the comparison of miRNA levels after tMCAO and RLIP induction. The y-axis corresponds to the mean expression value of log_10_ (p-value), and the x-axis displays the log_2_ fold change value. The blue dots represent the miRNA levels expressed in rat brain 24 h after stroke induction; the orange dots represent the miRNA levels expressed in rat brain 24 h after tMCAO + RLIP.

In order to identify those microRNAs that could be used as therapeutic targets for stroke, a miRNA expression profile in brain tissue affected by stroke and in the same brain region partly preserved by RLIP was performed. The whole miRNome of rats subjected to brain ischemia and to the neuroprotective protocol of remote limb conditioning was analysed in order to discriminate among miRNAs having an opposite trend of expression in the two experimental groups. As a visual tool useful to examine in an intuitive manner the expression profile of the 810 miRNAs analysed, a clustered heat map was depicted (Figure 1A). This map illustrated miRNA expression profiles across three experiment conditions: group A indicates sham-operated animals; group B indicates animals subjected to tMCAO; and group C indicates animals subjected to tMCAO + RLIP. From the general microarray, the heat map included only those miRNAs with p values less than 0.05. According to this criterion, many of the miRNAs selected showed a high level of expression in tMCAO and/or RLIP, in some cases, the expression pattern was significantly different between tMCAO and tMCAO + RLIP groups, both compared to sham-operated animals. In Figure 1 intensity values of detection for each miRNA are indicated in colour coded blocks with green being negative values, black being zero, and red being positive values, with brighter intensity to express higher levels. A negative value indicated downregulation and a positive value indicated an upregulation. In this way, those microRNAs whose regulation was higher or lower at least of 1.5 fold-changes (FC) (p < 0.05) if compared to the corresponding levels observed in sham-operated (FC ≤ 0,5 or FC > 1.5 vs. sham) were selected. This cut-off was set arbitrarily as a filter to select promising miRNAs to further confirm in a more sensitive method (RT-PCR). Examining the results obtained from microarray analysis, 21 microRNAs were selected for further analysis (Figure 1B) according to the following criteria: 1) an high signal intensity from in-depth data analysis (>1000, see methods section); 2) at least a 1.5-fold difference in expression between tMCAO and tMCAO + RLIP versus Sham-operated, respectively; 3) p-value < 0.05. The 21 selected miRNAs were the following: let-7a-5p, let-7b-5p, let-7c-5p, let-7d-5p, miR-15b-5p, miR-21-5p, miR-27b-3p, miR-32-3p, miR-34c-3p, miR-103-3p, miR-125a-5p, miR-126a-3p, miR-143-3p, miR-150-5p, miR-181c-5p, miR-191a-5p, miR-210-5p, miR-382-5p, miR-451-5p, miR-466b-5p and miR-485-3p.

### Stroke-induced upregulation of let-7a-5p, miR-143-3p, miR-451-5p and miR-485-3p was prevented by remote limb postconditioning

**Figure 2:**
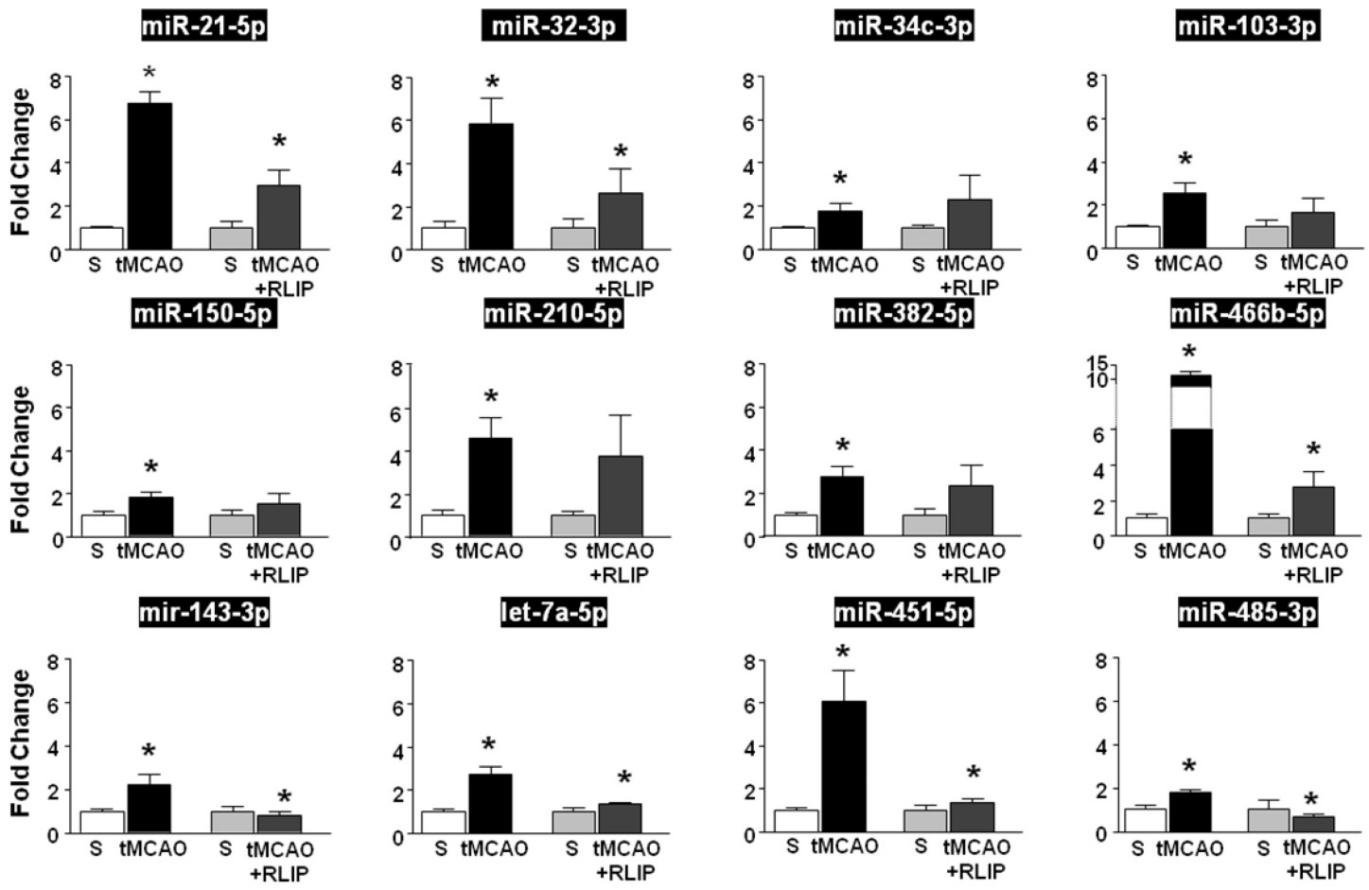
** Validation of microarray results by real-time PCR.** MicroRNA levels analysed by Real-Time PCR in ischemic brain regions from rats subjected to tMCAO and tMCAO + RLIP are expressed as fold change over the respective sham-operated controls. Each column represents the mean ± S.E.M. Results of microRNAs expression were normalized with respect to 4.5S RNA as internal control. n = 3 or 4 per group. S is for Sham-operated group.

The expression of miRNAs whose levels were significantly different among the experimental groups was validated by real-time PCR in the whole ischemic region of rats subjected to tMCAO and to RLIP. Among the 21 miRNAs selected through microarray analysis, RT-PCR revealed that 11 miRNAs (miR-21-5p, miR-32-3p, miR-34c-3p, miR-103-3p, miR-150-5p, miR-210-5p, miR-382-5p and miR-466-5p, let-7a-5p, miR-143-3p, miR-451-5p and miR-485-3p) were up-regulated in tMCAO group compared to tMCAO + RLIP and compared to sham-operated groups (Figure 2). The remaining 10 microRNAs (mir-15b-5p, miR-27b-3p, miR-125a-5p, miR-126a-3p, miR-181c-5p, miR-191a-5p, let-7b-5p, let-7c-5p, let-7d-5p) did not show any changes in RT-PCR (Figure S1).

In fact, among the 11 miRNAs that were upregulated after tMCAO, let-7a-5p, miR-143-3p, miR-451-5p and miR-485-3p were the only four miRNAs whose level of expression was restored after RLIP (Figure 2).

### Let-7a and miR-143 administration prevented neuroprotection elicited by remote limb postconditioning

The four selected miRNA mimics, let-7a, miR-143, miR-485 and miR-451, were intracerebroventricularly continuously administered for 48 h (9 μg/kg), starting 24 h before tMCAO + RLIP. This experimental protocol has been carried out in order to demonstrate that those miRNAs whose levels of expression were upregulated by stroke and restored by RLIP treatment could be involved in the neuroprotective state induced by RLIP.

**Figure 3:**
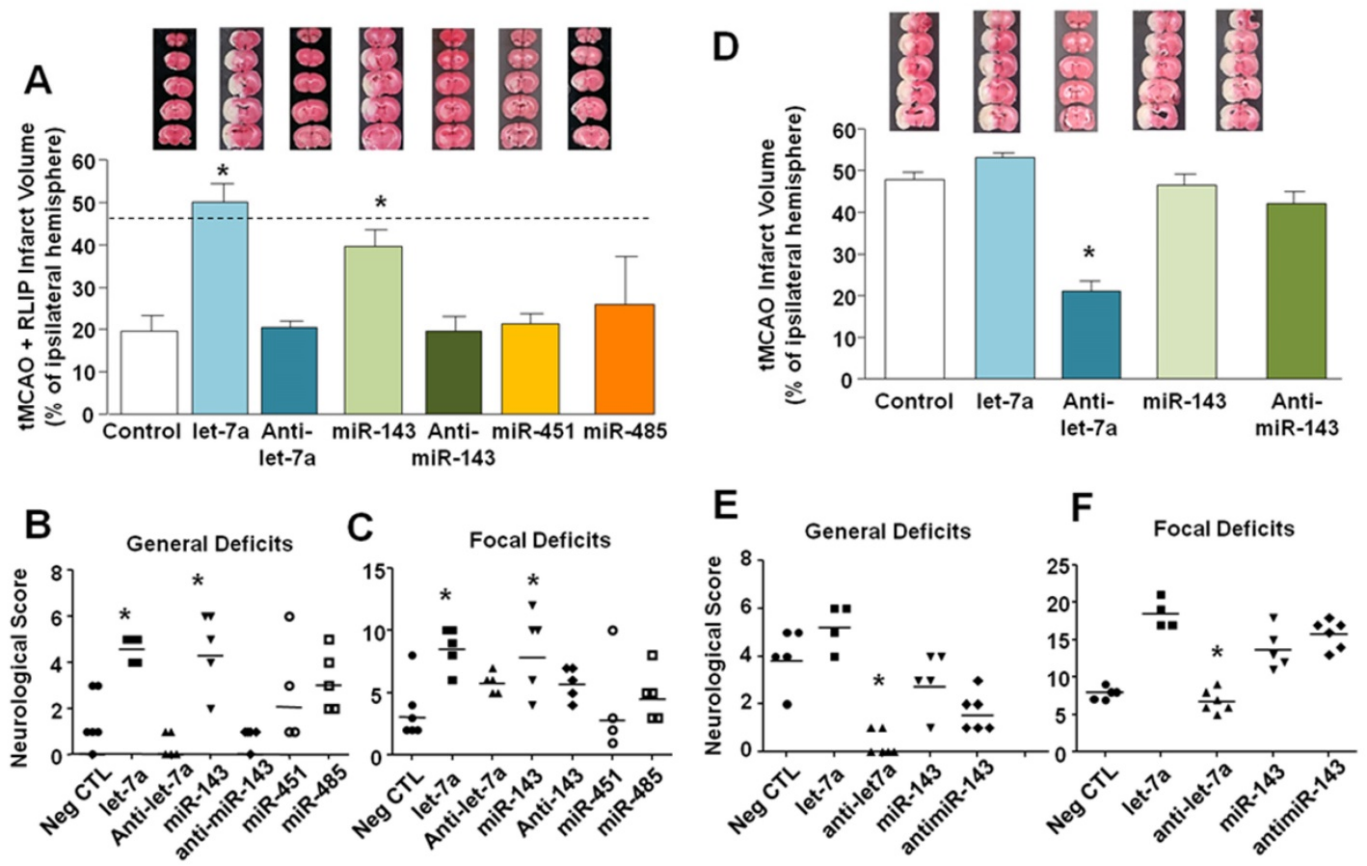
** In vivo effect of miRNAs administrations on ischemic volume and neurological scores of rats subjected to Remote Limb Ischemic Postconditioning or to tMCAO. (A)** Ischemic damage was assessed on rats subjected to continuous icv infusion of miRNA mimic (0,6 mg/kg) or Anti-miR (9 µg/kg) from 24 h before RLIP induction up to 24 h after reperfusion. On the top, representative pictures of brain sections comprising cortex and striatum are included. Brain damage is in white. Each column represents the mean ± S.E.M. n = 6 rats per tMCAO + RLIP + negative CTL group; n = 5 rats per tMCAO + RLIP + let-7a group; n = 6 rats per tMCAO + RLIP + Anti-let-7a group; n = 5 rats per tMCAO + RLIP + miR-143 group; n = 6 rats per tMCAO + RLIP + Anti-miR-143 group; n = 4 rats per tMCAO + RLIP + miR-485 group; n = 5 rats per tMCAO + RLIP + miR-451 group. *: p < 0,05 Vs tMCAO + RLIP + Negative CTL mimic. Dashed line indicates infarct volume referred to tMCAO animals. **(B-C)** Measurement of general and focal scores of neurological deficits of rats subjected to continuous icv infusion of miRNA mimic (0,6 mg/kg) or Anti-miR (9 µg/kg) from 24 h before tMCAO and RLIP induction up to 24 h after reperfusion. Each column represents the mean ± S.E.M. n = 6 rats per tMCAO + RLIP + negative CTL group; n = 5 rats per tMCAO + RLIP + let-7a group; n = 6 rats per tMCAO + RLIP + Anti-let-7a group; n = 5 rats per tMCAO + RLIP + miR-143 group; n = 6 rats per tMCAO + RLIP + Anti-miR-143 group; n = 4 rats per tMCAO + RLIP + miR-485 group; n = 5 rats per tMCAO + RLIP + miR-451 group. *: p < 0,05 Vs tMCAO + RLIP + Negative CTL mimic. **(D)** Ischemic damage was assessed on rats subjected to continuous icv infusion of miRNA mimic (0,6 mg/kg) or Anti-miR (9 µg/kg) from 24 h before tMCAO induction up to 24 h after reperfusion (Figure 4D). On the top, representative pictures of brain sections comprising cortex and striatum are included. Brain damage is in white. Each column represents the mean ± S.E.M. n = 5 rats per tMCAO + negative CTL group; n = 4 rats per tMCAO + let-7a group; n = 6 rats per tMCAO + Anti-let-7a group; n = 5 rats per tMCAO + miR-143 group; n = 6 rats per tMCAO + Anti-miR-143 group; *: p < 0,05 Vs tMCAO + negative CTL mimic. **(E-F)** Measurement of general and focal scores of neurological deficits of rats subjected to continuous icv infusion of miRNA mimic (0,6 mg/kg) or Anti-miR (9 µg/kg) from 24 h before tMCAO induction up to 24h h after reperfusion. Each column represents the mean ± S.E.M. n = 5 rats per tMCAO + negative CTL group; n = 4 rats per tMCAO + let-7a group; n = 6 rats per tMCAO + Anti-let-7a group; n = 5 rats per tMCAO + miR-143 group; n = 6 rats per tMCAO + Anti-miR-143 group; *: p < 0,05 Vs tMCAO + negative CTL mimic.

As shown in Figure 3, brain protection observed in rats subjected to RLIP was significantly lost when miR-let-7a or miR-143 mimics were icv infused (19.73 ± 3.76 in tMCAO + RLIP; 50.20 ± 4.30 after let-7a in tMCAO + RLIP, 20.52 ± 1.46, after Anti-let-7a in tMCAO + RLIP, 39.84 ± 3.99, after miR-143 in tMCAO + RLIP and 19.58 ± 3.67 after Anti-miR-143 in tMCAO + RLIP) (Figure 3A).

By contrast, treatment with miR-485 (25.92 ± 11.20) and miR-451 (21.30 ± 6.83) mimics did not revert neuroprotection elicited by RLIP (Figure 3A), thus suggesting that these two miRNAs were probably not involved in the neuroprotection induced by RLIP. In accordance with these data, loss of neuroprotection caused by administration of miR-let-7a and miR-143 was paralleled by a worsening of behavioural performances (general deficit scores in tMCAO + RLIP group 1.5 ± 0.5; in let-7a group 4.4 ± 0.4, in anti-let-7a group 0.4 ± 0.2, in miR-143 group 4.6 ± 0.75; in Anti-miR-143 0.8 ± 0.0; focal deficit scores in RLIP group 3.5 ± 0.96; in let-7a group 8.6 ± 0.75, in Anti-let-7a 5.8 ± 0.37, in miR-143 group 8.4 ± 1.47 and in Anti-miR-143 5.8 ± 0.84 ) (Figure 3 B-C). Treatment with miR-451 and miR-485 did not alter neurological scores if compared to controls (general deficits score in miR-451 group 3 ± 0.44, in miR-485 2.66 ± 1.66; focal deficit scores in miR-451 group 4.33 ± 0.51, in miR-485 4.33 ± 2.84). Sham RLIP was not included in the panel given that median ischemic volume and neurological deficits were comparable to those of Sham tMCAO group.

### Let-7a inhibition reduced stroke damage

To confirm that increase in let-7a and miR-143 expression induced by remote limb postconditioning exerted a neuroprotective effect, their respective mimics and antagomir (AntimiRNAs) were intracerebroventricularly continuously infused in tMCAO rats for 48 h (9 μg/kg), starting 24 h before surgical procedures (Figure 3D-E-F). As shown in panel D, Figure 3, only Anti-let-7a was able to completely recover ischemic damage induced by tMCAO (47,80 ± 1,80 in tMCAO group; 53.3 ± 0.91 after let-7a in tMCAO; 20.96 ± 2.43, after Anti-let-7a in tMCAO, 46.41 ± 2.88, after miR-143 in tMCAO and 42,16 ± 2.78, after Anti-miR-143 in tMCAO) (Figure 3D). This effect was accompanied by an amelioration of both general and neurological scores. Notably, increased levels of both miR-143 and let-7a during ischemia were not related to a worsening of the ischemic damage; this result was mirrored by a lack of significance in behavioural scores among the different experimental groups ( tMCAO+miR-143 vs. tMCAO and tMCAO+let-7a vs. tMCAO) (general deficit scores in tMCAO group 4.0 ± 0.55; in let-7a group 5.25 ± 0.47, in Anti-let-7a group 0.33 ± 0.6, in miR-143 group 3 ± 0.54 and in Anti-miR-143 group 1.66 ± 0.33; focal deficit scores in tMCAO group 8.0 ± 0.32; in let-7a group 8.6 ± 0.75 in let-7a group 18.5 ± 0.95, in Anti-let-7a group 6.83 ± 0.6, in miR-143 group 13.8 ± 1.24 and in Anti-miR-143 group 15.83 ± 0.79) (Figure 3E-F).

**Figure 4:**
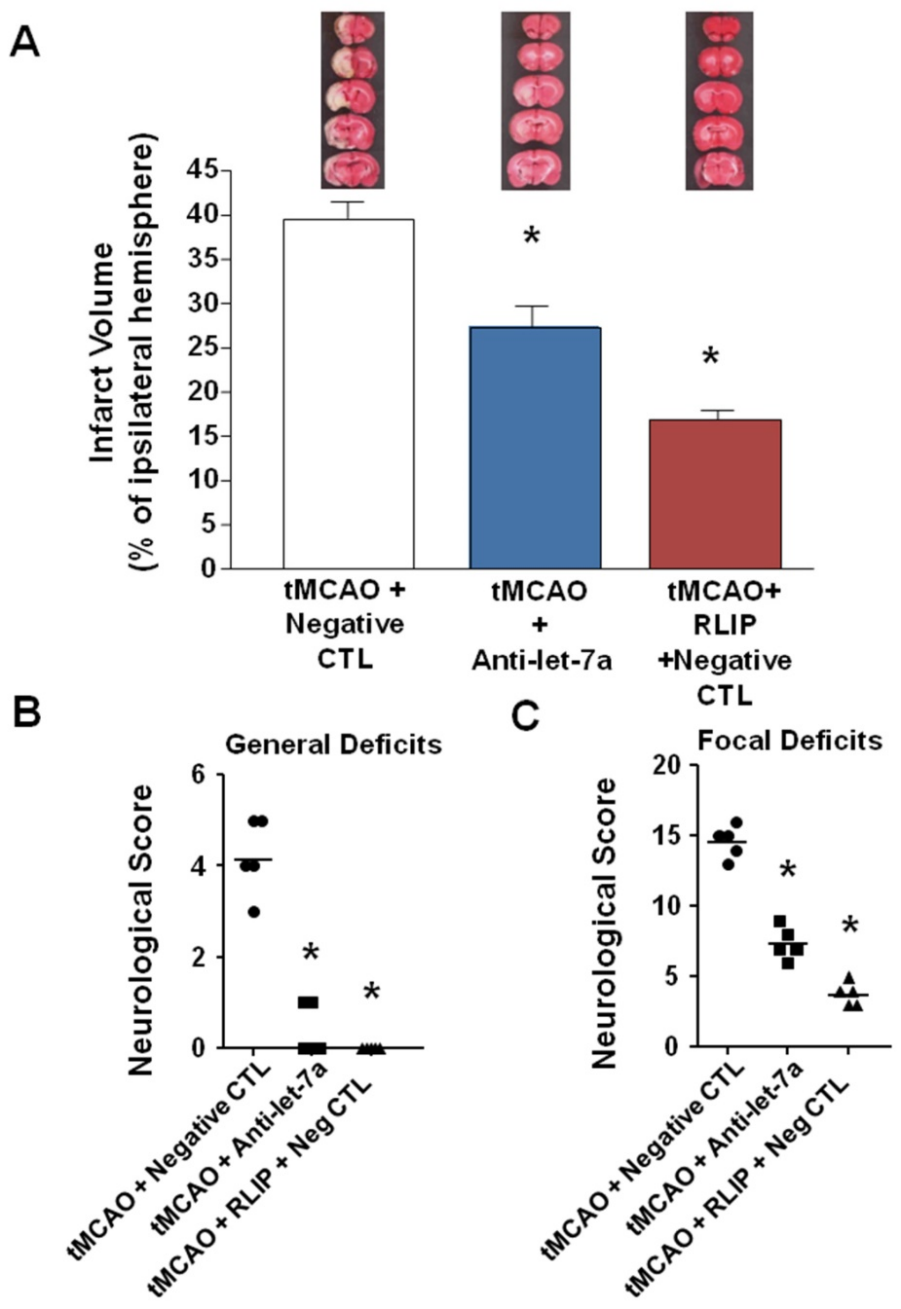
** Effect of Anti-let-7a administration on ischemic volume and neurological scores of rats subjected to tMCAO after 7 days of reperfusion. (A)** Ischemic damage was assessed on rats subjected to continuous icv infusion of Anti-miR (9 µg/kg) from 24 h before tMCAO induction up to 24 h after reperfusion and sacrificed 7 days after ischemia induction. On the top, representative pictures of brain sections comprising cortex and striatum are included. Brain damage is in white. Each column represents the mean ± S.E.M. n = 5 rats per tMCAO + negative CTL group; n = 5 rats per tMCAO + Anti-let-7a group; n = 5 rats per tMCAO + RLIP + negative CTL. **(B-C)** Measurement of general and focal scores of neurological deficits of rats subjected to continuous icv infusion of Anti-miR (9 µg/kg) from 24 h before tMCAO and tMCAO induction up to 24 h after reperfusion. Each column represents the mean ± S.E.M. n = 5 rats per tMCAO + negative CTL group; n = 5 rats per tMCAO + Anti-let-7a group; n = 5 rats per tMCAO + RLIP + negative CTL.

To test the hypothesis that Anti-let-7a could induce neuroprotection for an extended time window, antimiRNA was intracerebroventricularly infused for 48 h (9 μg/kg), starting 24 h before surgical procedures and rats sacrificed 7 days after ischemia induction. As shown in Figure 4A, Anti-let-7a was able to significantly reduce ischemic damage induced by tMCAO in a proportion similar to tMCAO + RLIP + Negative CTL group. (39.54 ± 1.9 in tMCAO + Neg CTL; 27.32 ± 2.28 in Anti-let-7a + tMCAO; 16.85 ± 0.57 in tMCAO + RLIP + Neg CTL) (Figure 4A). This effect was also mirrored by a specular variation of both general and neurological scores. General deficit scores 4.2 ± 0.37 in tMCAO + Neg CTL group, 0.4 ± 0.24 after Anti-let-7a in tMCAO group and 0.0 ± 0.0 in tMCAO + RLIP + Neg CTL; focal deficit scores 14.6 ± 0.5 in tMCAO + Neg CTL group, 7.4 ± 0.5 after Anti-let-7a in tMCAO group, 3.8 ± 0.37 in tMCAO + RLIP + Neg CTL) (Figure 4B-C).

### The upregulation of let-7a and miR-143 induced by stroke occurred both in NeuN and in GFAP positive astrocytes

**Figure 5:**
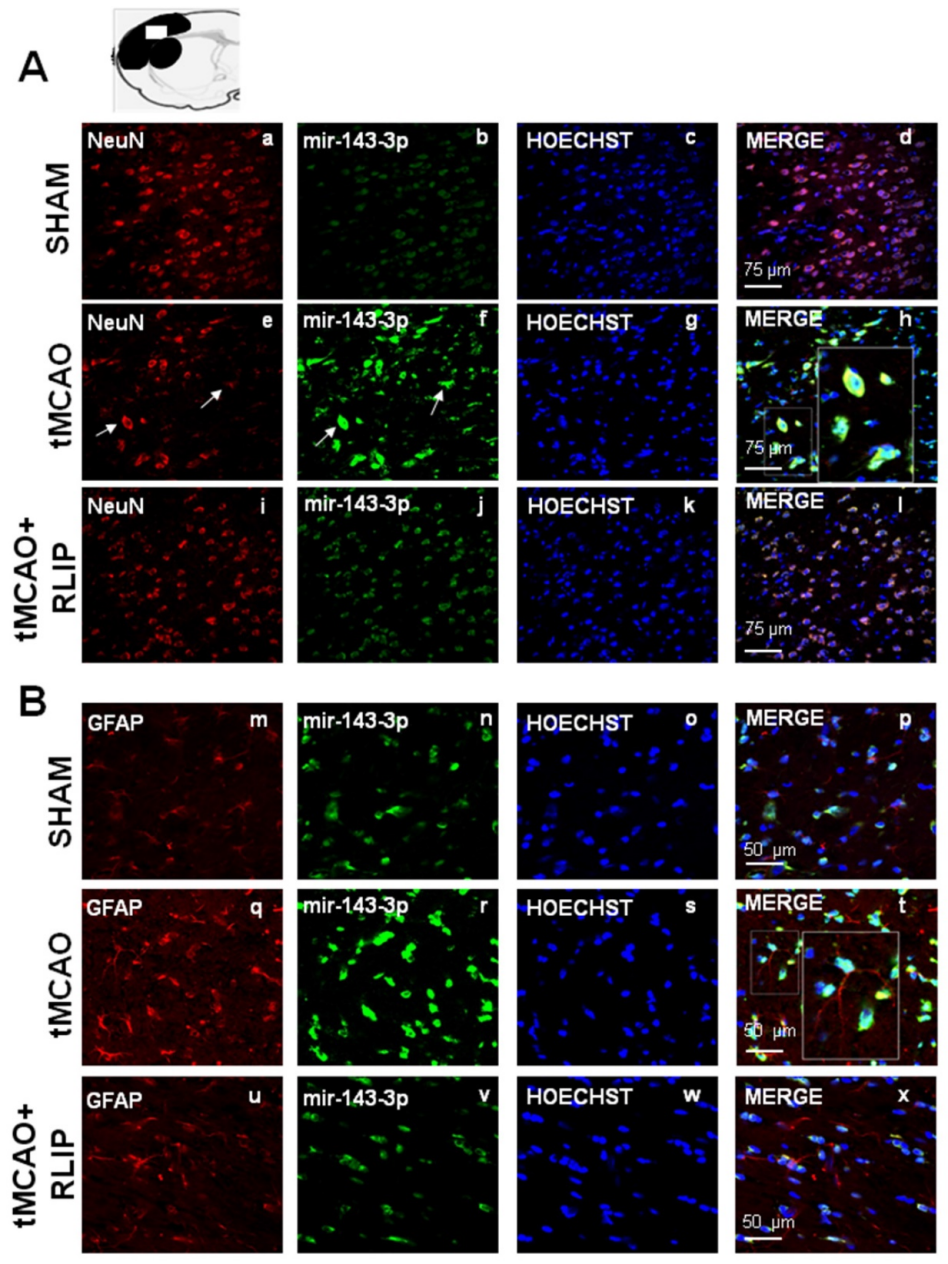
** Effect of 100 min of transient brain ischemia (tMCAO) and RLIP on miR-143 expression in cortical ischemic region. (A-I-M-U)** Confocal microscopic images displaying NeuN and GFAP in red, **(B-J-N-V)** Mir143-3p in green, **(C-K-O-W)** Hoechst in blue and **(D-L-P-X)** Merge in yellow in the brain ischemic regions of rats subjected to Sham-operation, tMCAO and tMCAO + RLIP. A representative brain slice cartoon indicating the area of interest is on the top of the Figure. Scale bars in A-L 75 μm in M-X 50 μm. High magnification zoom 2x in pictures H-T.

**Figure 6:**
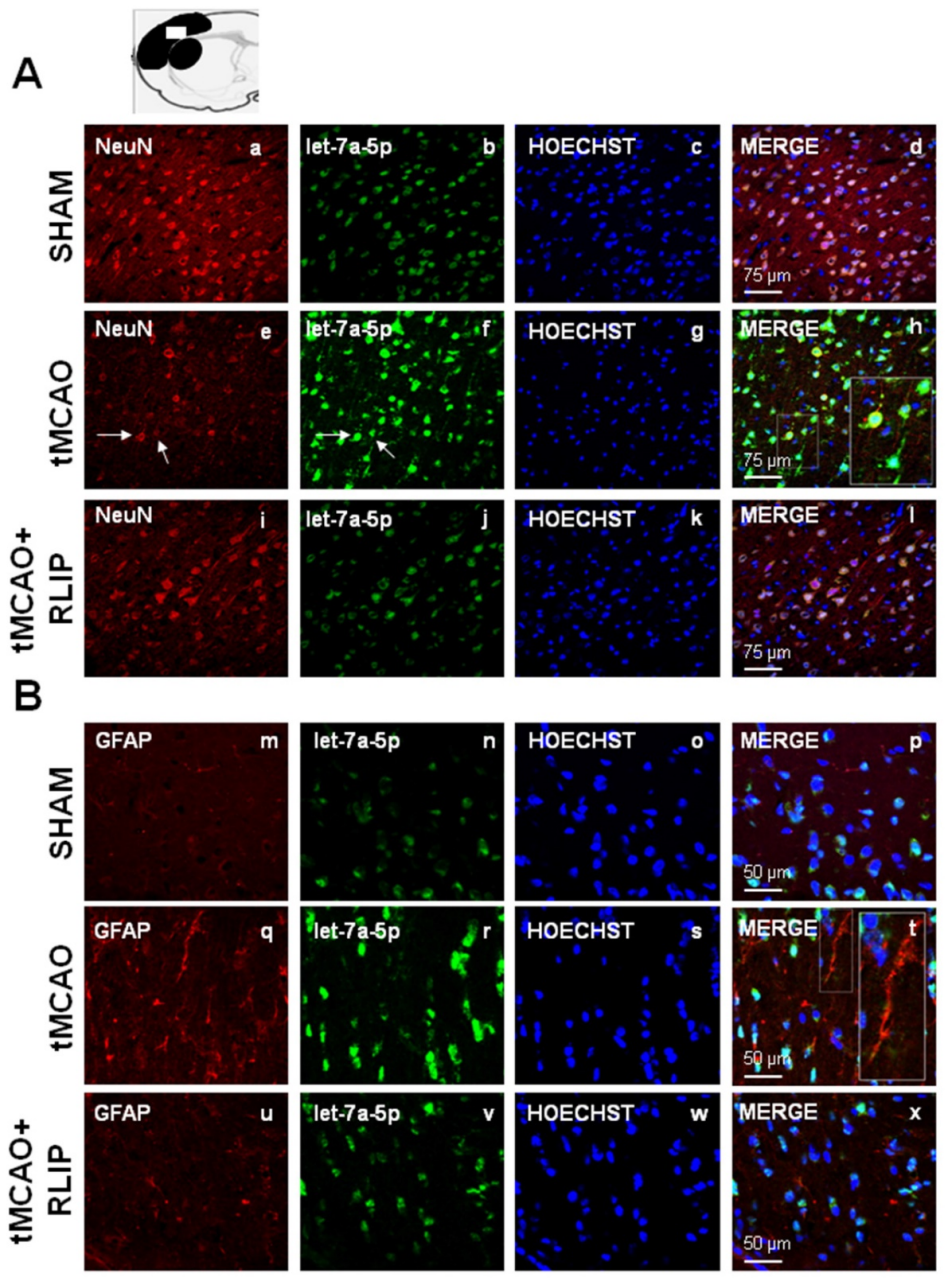
** Effect of 100 min of transient brain ischemia (tMCAO) and RLIP on let-7a expression in cortical ischemic region. (A-I, M-U)** Confocal microscopic images displaying NeuN and GFAP in red, **(B-J-N-V)** let-7a in green, **(C-K-O-W)** Hoechst in blue and **(D-L-P-X)** Merge in yellow in the brain ischemic regions of rats subjected to Sham-operation, tMCAO and tMCAO + RLIP. A representative brain slice cartoon indicating the area of interest is on the top of the Figure. Scale bars in A-L 75 μm in M-X 50 μm. High magnification zoom 2x in pictures H-T.

The expression of miR-143 and let-7a was evaluated by double fluorescence in situ hybridization with NeuN, neuronal marker and GFAP, astrocytic marker, in tissue slices from ipsilesional temporoparietal cortex of ischemic rats subjected to 100 min tMCAO and rats subjected to tMCAO + RLIP. Double fluorescence immunostaining with NeuN revealed that, in ischemic rats, robust miR-143 and let-7a immunoreactivity was localized mainly in the cytosol and in the nuclei of neurons (Figure 5E-H and Figure 6E-H). In particular, the expression of mir-143 and let-7a was very abundant in neurons undergoing morphological modification and degeneration that appeared close to death, as shown by the high magnification and the arrows in the Figures (Figure 5H and Figure 6H). Conversely, when the rats were subjected to tMCAO + RLIP, the immunoreactivity of miR-143 and let-7a decreased at a level comparable to that observed in Sham-operated rats (Figure 5A-D-I-L and Figure 6A-D-I-L). Furthermore, double fluorescence immunostaining with GFAP demonstrated that, in ischemic rats, the immunoreactivity of miR-143 was very rich in the nuclei of astrocytes while it seemed totally absent in branching astrocytes, as shown in high magnification of Figure 5Q-T. Differently from miR-143, the immunoreactivity of let-7a did not co-localize with the astrocytic marker GFAP, as shown in high magnification of Figure 6Q-T.

### Let-7a administration prevented HIF1- α overexpression induced by RLIP

In order to demonstrate the involvement of putative Let-7a targets on neuroprotection induced by remote postconditioning, the expression of HIF1-α , MKP1 and KRAS was evaluated in ischemic penumbra of rats subjected to tMCAO and to tMCAO + RLIP in presence of exogenous infused let-7a.

**Figure 7:**
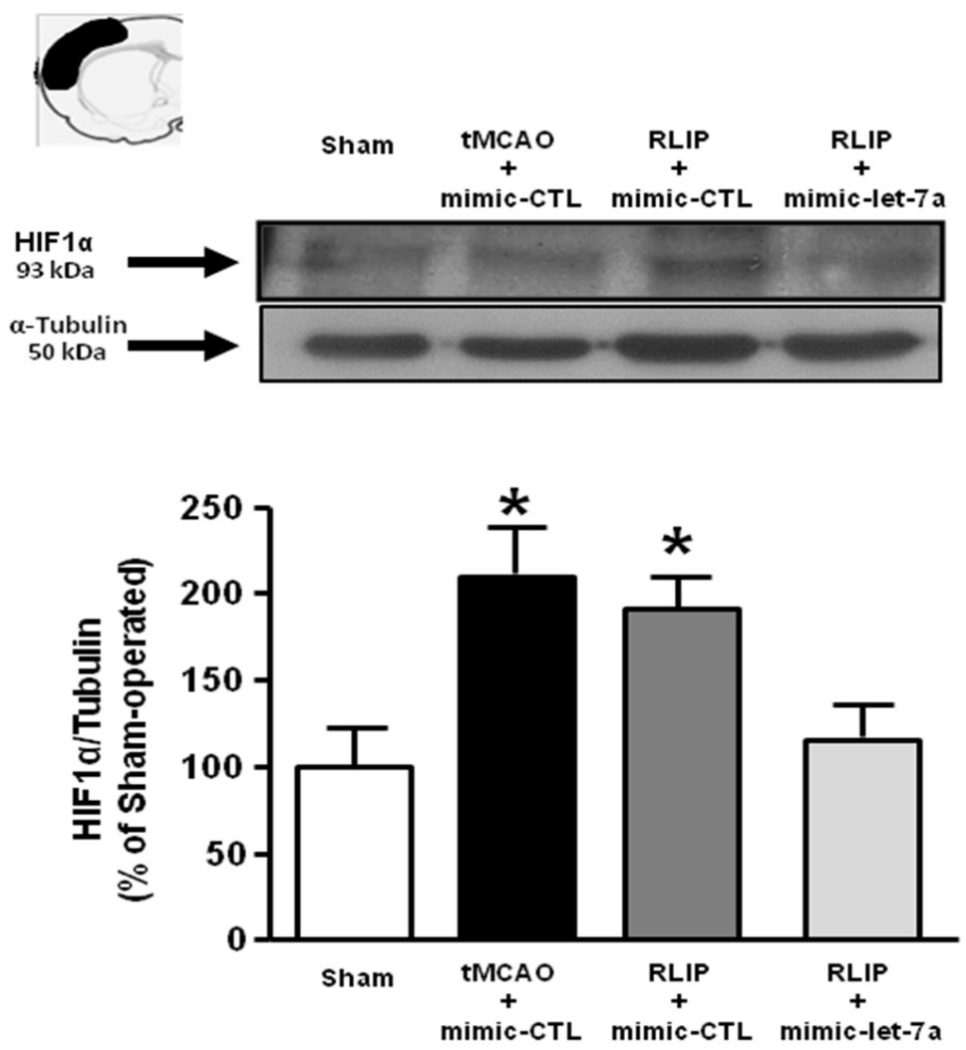
** Evaluation of HIF1α protein expression in tMCAO + RLIP rats upon mimic-let-7a-5p administration.** Evaluation of HIF-1α protein expression in ischemic rats subjected to remote limb postconditioning, intracerebroventricularly infused with mimic-let-7a-5p and sacrificed at 24 h from reperfusion. Protein levels are expressed as percentage versus the sham-operated controls. Each column represents the mean ± S.E.M. Results of protein expression were normalized with respect to α-tubulin. On the top of each graph, representative blots of HIF1α and α-tubulin signals are shown (A) HIF1α protein levels in cortex (n = 4 samples per sham group; n = 5 samples per tMCAO and RLIP groups; n = 4 samples per mimic-let-7a group). *: p < 0.05 vs. sham-operated controls.

Let-7a treatment was able to prevent HIF1-α overexpression induced by RLIP (Figure 7), while it did not affect the expression of the other two putative targets examined, MKP1 and KRAS (Figure S2).

## Discussion

In the present paper we identified for the first time a miRNA scripture of ischemic brain subjected to the protective strategy termed remote ischemic post-conditioning (RLIP). In particular, we were able to demonstrate that: (a) a specific miRNA signature characterizes the brain of ischemic animals subjected to tMCAO + RLIP; (b) the upregulation of let-7a and miR-143 occurring in the ischemic brain is prevented by ischemic remote postconditioning; (c) the neuroprotective effect of remote limb postconditioning is prevented by the icv administration of exogenous let-7a and miR-143; (d) icv administration of exogenous let-7a, but not miR-143, prevents HIF-1α upregulation induced by RLIP; (e) anti-let-7a reduces ischemic damage caused by tMCAO.

Remote limb ischemic postconditioning (RLIP) is a well-established neuroprotective strategy able to protect the brain from a previous harmful ischemic insult through a sub-lethal occlusion of the femoral artery. This strategy is being tested in clinical studies worldwide, nevertheless the mechanisms activated by RLIP and involved in the protection have not yet been completely elucidated 30.

In order to set up a pharmacological strategy able to recapitulate the neuroprotective phenotype induced by remote postconditioning, a specific miRNA subset, whose expression was modulated by RLIP induction and whose modulation may counteract stroke progression, has been identified. In this context the use of miRNA mimics or antimiRNA represents a very promising strategy in consideration of the fact that a single miRNA is able to regulate several proteins at the same time and that cerebral ischemia is a multifactorial pathology that in its progression involves several potential pharmacological targets 2.

The effect of brain conditioning on miRNA expression profiles of rat and mouse brain has been mainly evaluated by several studies carried out when the conditioning stimulus was applied before harmful ischemia, i.e. preconditioning 31,32. However, little is known about the involvement and the expression patterns of microRNA in remote ischemic conditioning. In the present study, among all 810 miRNAs examined in the brain by microarray microfluidic analysis, 21 microRNAs have been chosen to be validated by RT-PCR. In this preliminary analysis to select the most relevant miRNAs involved in RLIP-induced neuroprotection, no significant variation was observed between the sham operated animals and those subjected to the RLIP procedure without stroke. The diversified regulatory trend of a specific miRNA between the two experimental groups (tMCAO vs. tMCAO + RLIP) constituted the criterion of choice for the subsequent in vivo analyses. For instance, among 21 microRNAs, several of them were discarded because they were significantly upregulated in both tMCAO group and in tMCAO+RLIP group.

Thus, 4 miRNAs were further selected for their expression changes that resulted more significant compared to the others: let-7a-5p, miR-143-3p, miR-451-5p and miR-485-3p. Indeed, their levels strongly increased 24 h after ischemia induction, but were almost at pre-ischemic level when harmful ischemia was followed by remote ischemic postconditioning treatment.

Among the four miRNAs identified and selected, only let-7a and miR-143 might represent important targets for stroke therapy. These results are in agreement with expression data and functional experiments performed in other studies as well as with works on putative targets for these miRNAs 33,34. In fact, let-7a expression has previously already shown to be upregulated in brain tissue of rats subjected to tMCAO 33,34. Furthermore, let-7a gene knockdown has been demonstrated to protect against cerebral ischemia/reperfusion injury by inducing reduction of apoptosis and inflammatory reaction markers, as suggested by decreased number of p-p38 MAPK- and p-JNK-immunoreactive cells and by reduced levels of TNF-α and IL-6 after let-7a inhibitor treatment 35. In addition, K-RAS and Mitogen-Activated Protein Kinase Phosphatase 1 (MKP-1), which inactivates JNK1/2 and p38, have been recently shown to be targeted by let-7a 35, suggesting a possible role of this miRNA as mediator of neuroinflammation and apoptosis. Finally, in the field of ischemic stroke, HIF-1α represents another important target of let-7a 36. Among HIF-1α, MKP-1 and K-RAS, the ischemia-related let-7a putative targets examined in the present study, only HIF-1α seems to take part to the protective mechanism exerted by anti-let7a in brain ischemia and involved in remote postconditioning neuroprotection. In fact, the HIF-1α overexpression detected in the penumbra region of animals subjected to remote postconditioning was counteracted by the intracerebroventricular infusion of let-7a mimic, thus suggesting that the detrimental effect of let7a is partially mediated by the reduced expression of HIF-1α.

As regards miR-143-3p, predicted analysis indicates several membrane proteins involved in transduction pathways and solute carriers of different families as its probable targets. Hence, its role seems to be associated with cell responses to outer stimuli. Indeed, recent findings demonstrated the involvement of miR-143 in promoting mitochondria damage in cardiac ischemia, by targeting PKCε 37,38. However, in our experimental conditionins, although miRNA143 seems to be involved in remote postconditioning neuroprotection, nor the administration of miRNA143 mimic, nor anti-miRNA 143 modify the extent of infarct volume in ischemic rats.

In this regard, it is important to underline that, although the effect of these 2 miRNAs is occurring at neuronal levels, the expression of miRNA-143 was observed also in the body of astrocytic GFAP-positive cells, thus supporting the hypothesis that a different microRNA expression pattern may occur in astrocytes and neurons after ischemic injury 39. The peculiar expression of miRNA in neurons and glial cells has been already reported in other studies and several hypothesis have been postulated in order to explain this phenomenon 39-41, however further studies are needed in order to incontrovertibly support this hypothesis.

Overall, we demonstrated here that, after remote postconditioning, astrocytes and neurons express different miRNAs; this phenomenon may be due to the differences not only in their miRNA repertoire, but also in their cell specific function within the CNS. Therefore, we suggest that different miRNAs selectively expressed in the different CNS cells are involved in the protection induced by remote postconditioning in order for these cells to activate distinct biochemical pathways for better accomplishing their functions.

## Conclusions

Collectively, our data suggest that stroke neuroprotection conferred by RLIP is strongly prevented by let-7a and miR-143 mimic treatment. As consequence, these two miRNAs and their targeted proteins appear as potential mediators of the neuroprotection induced by RLIP. Future studies are required in order to identify, beside HIF1-α, which of the target proteins are involved in remote postconditioning-induced neuroprotection. Therefore, approaches able to regulate the expression levels of these miRNAs and their targeted proteins emerge as potential strategies in stroke intervention.

## Supplementary Material

Supplementary figures.

## Abbreviations

ECA: External Carotid Artery
FC: Fold Changes
GFAP: Glial Fibrillary Acidic Protein
MCA: Middle Cerebral Artery
tMCAO: transient Middle Cerebral Artery Occlusion
RIC: Remote Ischemic Conditioning
RLIP: Remote Limb Ischemic Postconditioning
