# Supplementary material for: Remote postconditioning ameliorates stroke damage by preventing let-7a and miR-143 up-regulation
Source: Theranostics. 2020 Oct 27;10(26):12174–88. doi: 10.7150/thno.48135 (PMC7667695; doi:10.7150/thno.48135)
Supplement: Supplementary file 1 — Supplementary figures. [file thnov10p12174s1.pdf]

SUPPLEMENTAL FIGURES LEGEND

S1

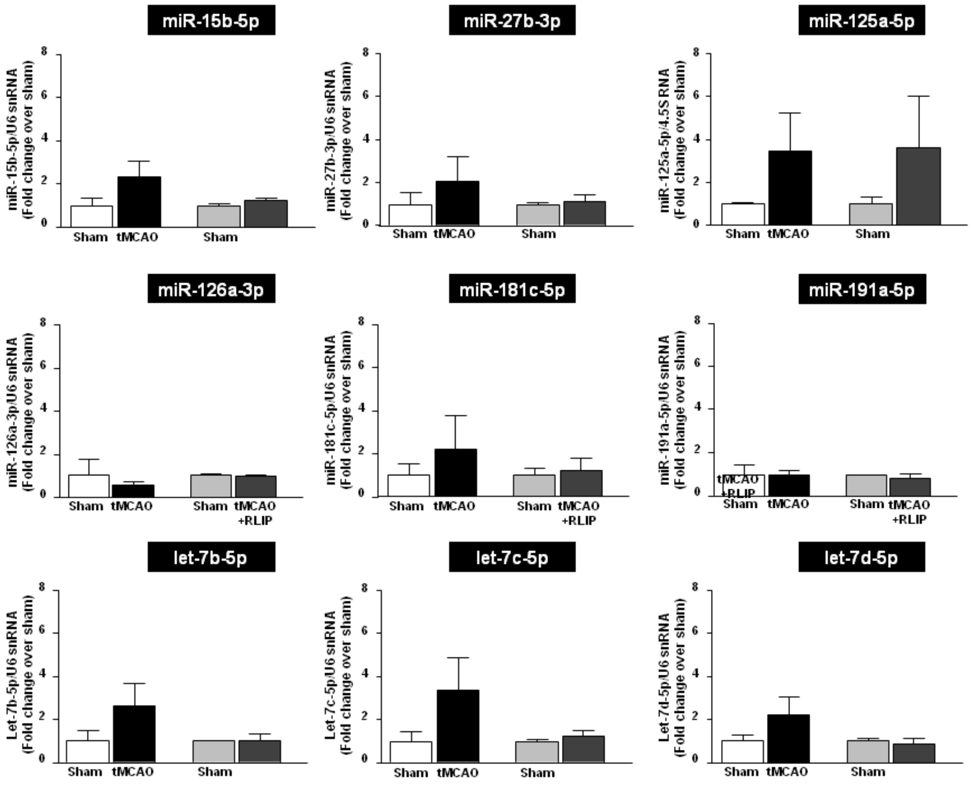

Figure S1. Completing 21 miRNA comparation after microarray. MicroRNA levels analysed by Real-Time PCR in ischemic brain regions from rats subjected to tMCAO and tMCAO + RLIP are expressed as fold change over the respective sham-operated controls. Each column represents the mean ± S.E.M. Results of microRNAs expression were normalized with respect to 4.5S RNA as internal control. n = 3 or 4 per group. *: $p < 0.05$ vs. Sham-operated group. #: $p < 0.05$ vs. tMCAO Group.

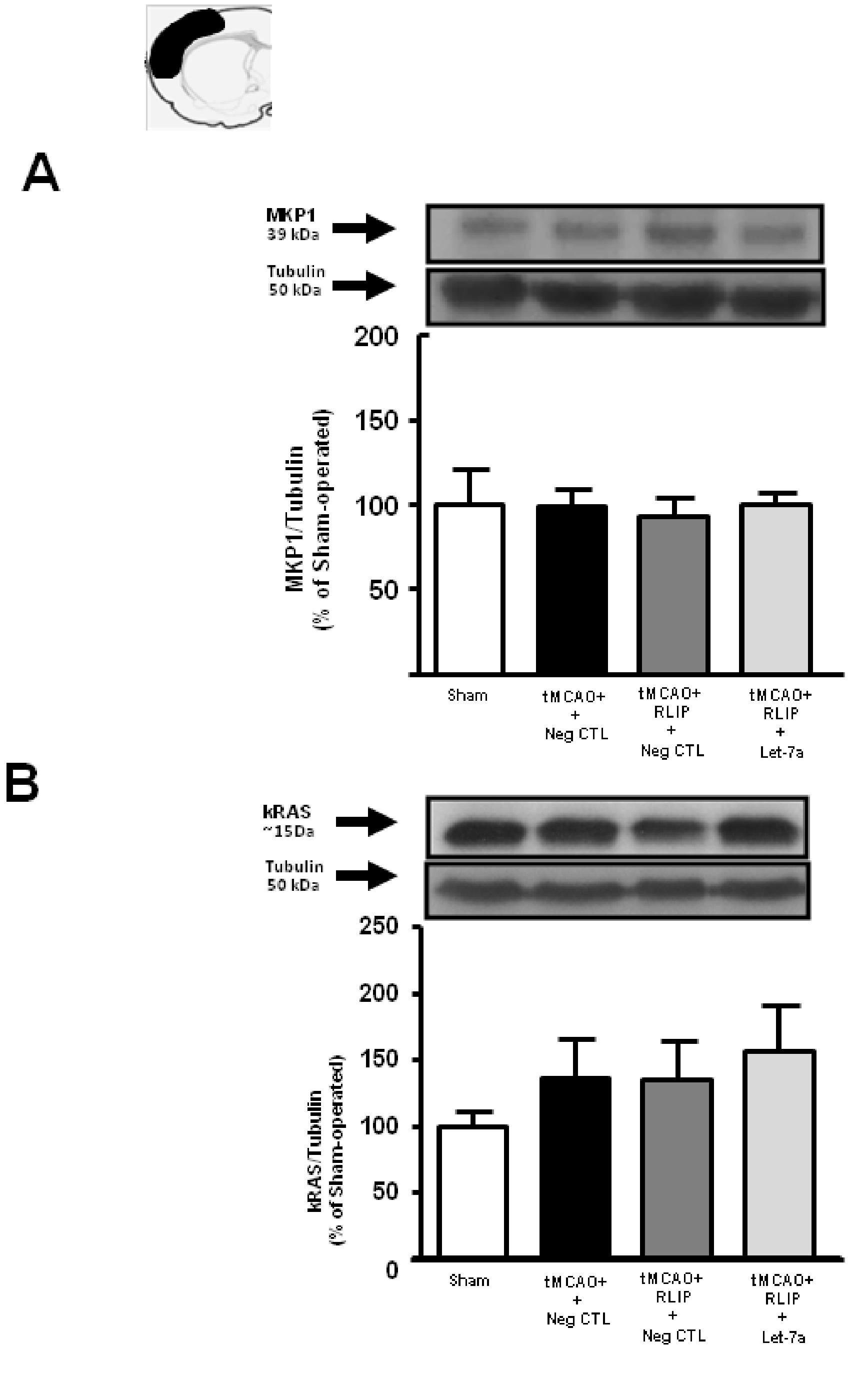
A
MKP1
39 kDa
Tubulin
50 kDa
200
150
100
50
MKP1/Tubulin
(% of Sham-operated)
Sham
tMCAO+
+
Neg CTL
tMCAO+
RLIP
+
Neg CTL
tMCAO+
RLIP
+
Let-7a
B
kRAS
~15Da
Tubulin
50 kDa
250
200
150
100
50
0
kRAS/Tubulin
(% of Sham-operated)
Sham
tMCAO+
+
Neg CTL
tMCAO+
RLIP
+
Neg CTL
tMCAO+
RLIP
+
Let-7a

Figure S2. Let-7a did not affected protein expression of KRAS and MKP1

Evaluation of MKP1 and kRAS protein expression in ischemic rats subjected to remote limb postconditioning, intracerebroventricularly infused with mimic-let-7a-5p and sacrificed at 24 h from reperfusion. Protein levels are expressed as percentage versus the sham-operated controls. Each column represents the mean ± S.E.M. Results of protein expression were normalized with respect to α-tubulin. On the top of each graph, representative blots of MKP1 and kRAS and α-tubulin signals are shown (A) MKP1 protein levels in cortex (n = 4 samples per each group). (B) kRAS protein levels in cortex (n = 4 samples per each group). *: $p < 0.05$ vs. sham-operated controls.
